# Oxidative Stress in Calcific Aortic Valve Stenosis: Protective Role of Natural Antioxidants

**DOI:** 10.3390/antiox11061169

**Published:** 2022-06-14

**Authors:** Radhika Adhikari, Saugat Shiwakoti, Ju-Young Ko, Bikalpa Dhakal, Sin-Hee Park, Ik Jun Choi, Hyun Jung Kim, Min-Ho Oak

**Affiliations:** 1 College of Pharmacy and Natural Medicine Research Institute, Mokpo National University, 1666 Yeongsan-Ro, Cheonggye-Myeon, Muan-Gun 58554, Jeonnam, Korea; radhika@mokpo.ac.kr (R.A.); saugat.shiwakoti04@mokpo.ac.kr (S.S.); herolegend@mokpo.ac.kr (J.-Y.K.); bklpdhakal@mokpo.ac.kr (B.D.); hyunkim@mokpo.ac.kr (H.J.K.); 2 Catholic Research Institute for Intractable Cardiovascular Disease (CRID), College of Medicine, The Catholic University of Korea, Seoul 06591, Korea; sinhee@catholic.ac.kr (S.-H.P.); mrfasthand@catholic.ac.kr (I.J.C.)

**Keywords:** calcific aortic valve stenosis, valvular interstitial cells, oxidative stress, reactive oxygen species, antioxidant

## Abstract

Calcific aortic valve stenosis (CAVS) is the most prevalent heart valvular disease worldwide and a slowly progressive disorder characterized by thickening of the aortic valve, calcification, and subsequent heart failure. Valvular calcification is an active cell regulation process in which valvular interstitial cells involve phenotypic conversion into osteoblasts/chondrocytes-like cells. The underlying pathophysiology is complicated, and there have been no pharmacological treatments for CAVS to date. Recent studies have suggested that an increase in oxidative stress is the major trigger of CAVS, and natural antioxidants could ameliorate the detrimental effects of reactive oxygen species in the pathogenesis of CAVS. It is imperative to review the current findings regarding the role of natural antioxidants in CAVS, as they can be a promising therapeutic approach for managing CAVS, a disorder currently without effective treatment. This review summarizes the current findings on molecular mechanisms associated with oxidative stress in the development of valvular calcification and discusses the protective roles of natural antioxidants in the prevention and treatment of CAVS.

## 1. Introduction

As per the World Health Organization, cardiovascular diseases are considered a leading cause of death globally. In 2019, an estimated 17.9 million people died from cardiovascular diseases, representing 32% of total global deaths [1]. Calcific aortic valve stenosis (CAVS) is currently considered the third most prevailing cardiovascular disease after coronary artery disease and hypertension [2]. Research showed that approximately 2–4% of people aged over 65 would develop CAVS with an 80% risk of development of heart failure, aortic valve replacement, or death after five years of disease progression [3].

CAVS is a slowly progressive disorder characterized by thickening of the aortic valve, fibrosis, and calcification of valve leaflets, leading to complete obstruction of the left ventricular outflow [4]. CAVS requires proper diagnosis and treatment; if not treated, it leads to cardiac hypertrophy and, eventually, heart failure. The only available treatment is aortic valve replacement, however, with limitations, such as permanent use of anticoagulants and the need for reoperation due to the limited lifespan of the prosthetic valve [5,6]. So, it is necessary to find a novel therapeutic strategy, that is only possible through the elucidation of the molecular mechanisms of CAVS [7]. At present, there is emerging evidence of a critical role of oxidative stress in the initiation and progression of CAVS [8,9]. Furthermore, studies have shown the importance of natural antioxidants in ameliorating the detrimental effects of reactive oxygen species (ROS) in halting the progression of CAVS. Therefore, in this review, we discuss the pathophysiology of CAVS associated with oxidative stress and the role of antioxidants as potential therapeutic candidates for CAVS prevention and management.

## 2. Clinical Risk Factors and Treatment of CAVS

There are various risk factors associated with the development of CAVS, which fall mainly into three groups: patient-related, hemodynamic-related, and valve-related [10]. Patient-related risk factors include older age, smoking, hypertension, obesity/diabetes, lipid abnormalities, chronic renal failure, and concomitant coronary artery disease. Hemodynamic-related risk factors include left ventricular systolic dysfunction and/or low cardiac output, hemodynamic changes during exercise, and atherosclerosis that leads to faster progression of CAVS. Similarly, the valve-related risk factor mainly includes a bicuspid valve, a congenital risk factor for CAVS [10]. Studies have shown the above-mentioned risk factors for CAVS incidence, but there are more to explore. Oxidative stress is directly or indirectly associated with several risk factors, such as lipid abnormalities, obesity, diabetes, atherosclerosis, smoking, aging, etc. [11,12,13,14,15,16]. Thus, oxidative stress is being proposed as the unifying mechanism for many CAVS risk factors. The proper management of all the risk factors at an early stage can effectively prevent the incidence of CAVS. However, because some of the risk factors cannot be managed entirely, an effective approach to assess the risk factors and implementation for CAVS prevention is needed.

To date, the only treatment option for severe CAVS is surgical valve replacement or transcatheter intervention [17]. Aortic valve replacement is the surgical removal of the calcified valve and replacement with a biological valve. A mechanical valve is implanted in symptomatic severe aortic stenosis patients with a small aortic annulus [18]. Surgical aortic valve replacement improves the patient’s survival rate by improving symptoms, but it is a very invasive procedure, and various complications occur after valve replacement, such as acute renal failure, atrial fibrillation, and high blood transfusion rates [19]. Transcatheter aortic valve replacement is a less invasive procedure that uses a catheter to replace a calcified valve. Recent studies reported that the mortality rate after transcatheter valve replacement is lower than that of surgical valve replacement [20]. However, complications arise, such as a high residual aortic regurgitation rate and the need for pacemaker implantation [19].

Both valve replacement procedures give rise to the most common problem—prosthetic mismatch—which can lead to bioprosthetic valve dysfunction, diminished regression of the left ventricular mass, symptoms recurrence, and unfavorable clinical outcomes [21]. Therefore, it is crucial to find treatment alternatives to alleviate aortic valve stenosis due to various risk factors and complications.

## 3. Oxidative Stress in the Pathophysiology of CAVS

**Figure 1:**
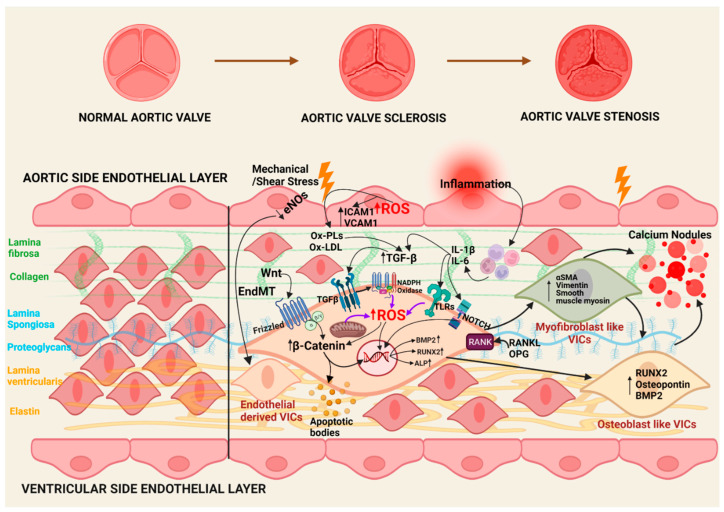
Illustrative diagram of the pathophysiology of CAVS. BMP—bone morphogenic protein, eNOS—endothelial Nitric oxide synthase, ICAM-1—intercellular adhesion molecule 1, IL—interleukin, NF-κB—nuclear factor κB, OPG—osteoprotegerin, Ox-LDLs—oxidized low-density lipoproteins, Ox-PLs—oxidized phospholipids, RANK—receptor activator of nuclear factor kappa B, RANKL—RANK ligand, ROS—reactive oxygen species, Runx2—runt-related transcription factor 2, TGF—transforming growth factor, TLRs—Toll-like receptors, VCAM-1—vascular cell adhesion protein 1, VICs—valvular interstitial cells, Wnt—wingless and Int, α-SMA—α-smooth muscle actin.

The pathophysiology of CAVS is complex; however, increasing evidence points to the critical role of oxidative stress in the initiation and propagation phases of CAVS (Figure 1) [8,9]. Understanding these roles requires a brief overview of the cellular mechanisms driving CAVS, which are briefly discussed below. The onset and progression of calcific aortic valve stenosis are considered two stages. In the initiating stage, there is endothelial dysfunction and lipid deposition, and in the progressive stage, inflammation, fibrosis, and calcification occur at the end.

### 3.1. Initiation Phase

The initial phase of calcification is initiated by endothelial dysfunctions of valvular endothelial cells, triggered by various risk factors, such as mechanical/shear stress, lipid deposition, ROS, and inflammation [22].

Mechanical/shear stress facilitates lipoprotein infiltration, specifically low-density lipoprotein (LDL) and Lp(a), and endothelial to mesenchymal transition (EndMT) to form endothelial-derived VICs, which undergo osteoblastic differentiation and calcification [22,23]. Additionally, the activation of Notch signaling in these cells increases TGF β, which leads to the weakening of the endothelial barrier function promoting EndMT [24]. This further induces the Wnt/β-catenin signaling pathway to increase calcification [25,26,27].

Multiple studies conducted in explanted calcified aortic valve leaflets indicate dysregulation of endothelial nitric oxide synthase (eNOS) pathway and increased nicotinamide adenine dinucleotide phosphate (NADPH) oxidase levels leading to oxidative stress [28,29,30]. This increased oxidative stress increases the formation of oxidized low-density lipoproteins (Ox-LDLs) and oxidized phospholipids (Ox-PLs) that act as a stimulus to initiate calcification [16]. These oxidized lipoproteins cause increased adherence and extravasation of immune cells by upregulating cell adhesion molecules, such as intercellular adhesion molecule 1 (ICAM-1) and vascular cell adhesion protein 1 (VCAM-1) [30,31,32,33].

Another stimulus is the deposition of lipoproteins, leading to chronic inflammation, which is considered a significant characteristic of the early stage of CAVS [34]. Inflammation activates the uptake of oxidized lipids, which induces the release of cytokines and activates TLRs (Toll-like receptors), TGF-β, and NOTCH signaling pathways to induce differentiation of cells [22,35,36]. Furthermore, monocytes and macrophages induce osteogenic differentiation of VICs and calcification via secretion of tumor necrosis factor (TNF), and then, activation of nuclear factor kappa B (NF-κB) and interleukin (IL)-1β and IL-6 [37,38,39,40]. 

Thus, ROS produced from various sources is responsible for the initiation as well as propagation phase to induce uptake of oxidized lipoproteins and activate multiple pathways and β-catenin accumulation causing osteoblastic differentiation and calcification.

### 3.2. Propagation Phase

After completion of the initial inflammation phase, the propagation phase starts with the differentiation of valvular interstitial cells (VICs) into myofibroblastic and osteoblastic phenotype, which is regulated by cytokines secreted by immune cells [41]. VICs are widely distributed throughout the three layers of leaflets in the aortic valve and have a significant role in the progression of CAVS [3,42]. VIC differentiation induced by cytokines increases alkaline phosphatase activity and expression of several osteoblast markers, such as runt-related transcription factor 2 (Runx2), bone morphogenic protein 2 (BMP2), and osteopontin (OPN) [43].

After initiation of calcification and slow progression of disease toward the later stage of calcification, various calcific pathways are responsible for calcification induction. These pathways include Notch signaling pathways, receptor activator of nuclear factor kappa B (RANK/RANK ligand/osteoprotegerin (OPG) pathway, and wingless and Int-1 (Wnt)/β-catenin [2,44].

The notch signaling pathway is responsible for repressing BMP2 expression and Runx2 transcriptional activity preserving VICs from osteoblastic differentiation and calcification. However, when this pathway is impaired during the propagation phase, there will be a higher expression of BMP2 and Runx2 transcriptional activity [45]. Furthermore, it upregulates (Wnt)/β-catenin and increases RANK/ RANK ligand interactions. In human VICs, RANKL binding can lead to osteoblastic differentiation, which is counteracted by OPG, as evidenced by the study indicating an increased level of RANKL and decreased level of OPG in calcified aortic valves [46].

Wnt binds to the LDL receptor-related protein 5 (LRP5) and activates the Wnt/β-catenin pathway, a positive regulator of osteoblastic differentiation [47]. This pathway is further activated by increased mechanical strain and injury due to the stiffening of cusps by calcium deposition [48]. This increases osteoblastic differentiation of VICs through ectonucleotide pyrophosphatase/phosphodiesterase 1(ENPP 1). In addition, ENPP1 promotes further calcification by generating adenosine triphosphate (ATP) and inorganic phosphate. Additionally, apoptosis in VICs occurs due to the loss of ATP [48], as ATP plays a role in increasing survival signaling in VICs, promoting BMP2 expression, and increases calcification of VICs [48,49].

### 3.3. Oxidative Stress in CAVS

An imbalance between reactive oxygen species (ROS) production and cellular antioxidant capacity results in oxidative stress, which plays a vital role in the pathogenesis of various cardiovascular diseases, including endothelial dysfunction, hypertension, vascular calcification, atherosclerosis, cardiac remodeling, stroke, and diabetes [50,51]. These highly reactive intermediates are mainly produced in mitochondria. In addition to the mitochondrial system, there are other sources of ROS, such as membrane NAD(P)H oxidase (NOX), xanthine oxidases, and nitric oxide synthase (NOS) [52]. NOX is responsible for the transfer of electrons across the plasma membrane and the generation of superoxide from NADPH [50,52]. Xanthine oxidase is responsible for catalyzing the oxidation of hypoxanthine to xanthine using oxygen and releasing superoxide anions and H_2_O_2_. NOS acts as a dimer to catalyze the formation of nitric oxide (NO), whose uncoupling results in superoxide generation, as the enzyme functions switch from NOS to NADPH-dependent oxidase. The generated superoxide reacts with NO to reduce the availability of NO, resulting in increased pressure overload, left ventricular hypertrophy, and diastolic dysfunction. Eventually, this causes an increase in ROS and damages the heart cells.

To inactivate the generated ROS and prevent its damage, biological systems consist of defense antioxidant mechanisms, which are divided into two types, namely primary enzymatic antioxidant system and non-enzymatic antioxidant system [53]. Primary enzymatic antioxidant system consists of various enzymes, such as superoxide dismutase, catalase (CAT), glutathione perioxidase (GPx), and DT- diaphorase, and non-enzymatic compounds include bilirubin and albumin [53]. To prevent ROS-induced oxidative stress, antioxidant enzymes, such as SODs, bind O_2_^−^ to an oxidized form of the enzyme, which, after acquiring another proton, releases molecular oxygen and catalase breakdown H_2_O_2_ into molecular oxygen and water [54].

An increase in activity of NAD(P)H oxidase, “uncoupling” of NOS, and maladaptive changes in the expression of antioxidants lead to oxidative stress in cardiovascular diseases [55]. Studies have also suggested that in the calcified aortic valve, the “uncoupling” of NOS leads to an increase in oxidative stress of calcified valves by peroxide formation and a decrease in Super-oxide Dismutases (SODs) [55]. Along with total SODs activity, antioxidant enzymes, such as copper-zinc superoxide dimutase (SOD1), manganese SOD (SOD2), or extracellular SOD (SOD3) and catalase, are reduced in calcific lesions, leading to oxidative stress due to increase in ROS [29]. Moreover, it has been shown that xanthine oxidase could increase oxidative stress and promote osteoblast differentiation of vascular smooth muscle cells [52]. 

In isolated cultured porcine VICs, P38 MAPK and MEK1/2/ERK1/2 pathways are involved in calcium nodule formation by TGF-β1-induced ROS production [56]. ROS is also associated with osteogenic differentiation via Nox2-mediated GKS3β/β-catenin in VICs [57]. Similarly, DNA damage associated with ROS production has shown to upregulate osteogenic transcription factors, such as Runx2 via AKT activation, leading to an increase in osteogenic differentiation [58]. Previous studies have shown that an increase in ROS in calcified aortic valves induces oxidative stress associated with intracellular inflammation [9].

Thus, during the pathogenesis of CAVS, increased formation of ROS acts in the initiation as well as propagation phase of CAVS. In the initiation phase, a pool of ROS generated from various sources increases the oxidative and inflammatory responses of infiltrating lipids. During the propagation phase, ROS activates various signaling pathways, including p38, ERK 1/2, AKT, GKS3β/β-catenin, and NFκβ, which in turn increases the expression of fibrotic and osteogenic genes, leading to VICs differentiation to osteoblastic phenotype.

## 4. Protective Effect of Natural Antioxidants on CAVS Development

The reduction in the level of antioxidant enzymes has been shown to increase the gene expression of Runx2 and OPN in isolated human VICs. The hVICs derived from calcified valves were more susceptible to oxidative stress and lacked antioxidant defense mechanisms [59]. Owing to the role of oxidative stress in the pathophysiology of CAVS, targeting the inhibition of ROS with antioxidants can be a promising therapeutic approach for the treatment and prevention of CAVS. Antioxidants are known to retard autoxidation by inhibiting free radical formation or by enhancing the activity of intracellular antioxidant enzymes. The mechanism of free radical inhibition and generation occurs by different mechanisms, which include: (a) scavenging peroxidation initiating species, (b) chelating metal ions to make them unable to generate reactive species or decompose lipid peroxides, (c) quenching ^•^O_2_^−^ preventing the formation of peroxides, (d) breaking the autoxidative chain reaction, and/or (e) reducing localized O_2_ concentrations [60].

The increased oxidative stress and ROS generation can also be ameliorated by increasing the intake of antioxidants exogenously [35]. The exogenous antioxidants source includes food and medicinal plants, namely vegetables, fruits, flowers, spices, mushrooms, beverages, and traditional medicinal herbs [61]. Studies show that plant serves as a major source of antioxidants [62]. The natural antioxidants obtained from plant materials are mainly polyphenols (phenolic acids, flavonoids, anthocyanins, lignans, and stilbenes), carotenoids (xanthophylls and carotenes), and vitamins (vitamin E and C). Various natural compounds are known to have antioxidant effects; among them, flavonoids are considered potent antioxidants [61].

**Figure 2:**
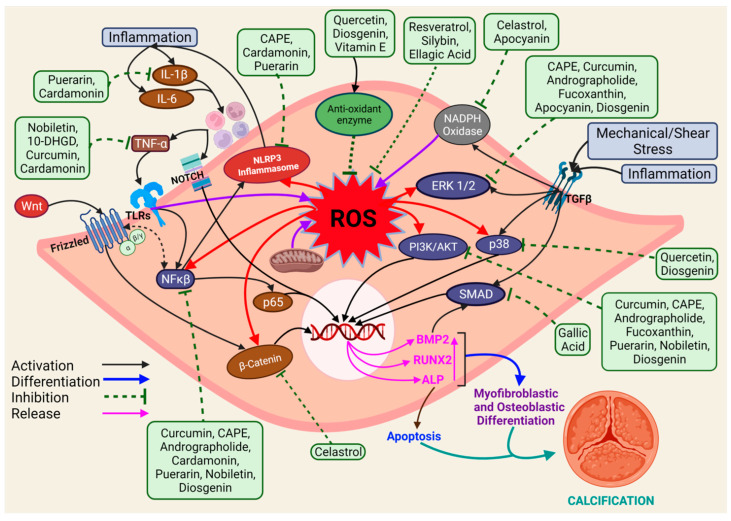
A diagram showing antioxidants with their mechanism of inhibition of CAVS. ROS is generated by different factors in cells, which can further activate various signaling pathways, leading to the release of transcription factors responsible for causing CAVS. Green rectangular boxes are antioxidants. The brown oval and the rectangular box indicate factors released during pathogenesis. The blue oval box represents signaling pathways. The red oval box indicates proteins. The pink text indicates proteins responsible for cellular differentiation. Red arrows indicate factors activated by ROS, and purple arrows indicate factors activating ROS. 10-DHGD—10 dehydrogingerdione, AKT —Protein Kinase B, ALP- Alkaline phosphatase, BMP—bone morphogenic protein, CAPE—Caffeic acid phenethyl ester, ERK—Extracellular signal-regulated kinase, IL—interleukin, NF-κB—nuclear factor κB, NLRP3—NLR family pyrin domain containing 3, ROS—reactive oxygen species, Runx2—runt-related transcription factor 2, SMAD—small mothers against decapentaplegic, TGF—Transforming Growth Factor, TLRs—Toll-like receptors, TNF—tumor necrosis factor, VICs—valvular interstitial cells, Wnt—wingless and Int.

In recent years, natural antioxidants have gained considerable attention for their protective role in CAVS. We tried to summarize the progress of natural antioxidants studied for CAVS, which might be beneficial for potential therapeutic implications in the near future. The list of natural antioxidants that are known to prevent CAVS is presented in Table 1, and the antioxidants with their mechanism of inhibition of CAVS are shown in Figure 2. 

### 4.1. Curcumin

Curcumin (1,7-bis (4-hydroxy-3-methoxyphenyl)-1,6-heptadiene-3,5-dione) or diferuloylmethane is the main natural bioactive constituent of *Curcuma longa* (turmeric), a natural herb traditionally used in Asian countries as a spice [82]. It is identified as a potential candidate for treating inflammatory diseases, cancer, cardiovascular diseases, and others [63,82,83]. It exerts cardiovascular protective actions among cardiovascular diseases by mitigating oxidative stress by preventing Ox-LDL formation [84] and improving serum lipids levels [85]. In hVIC, curcumin inhibited osteogenic differentiation by interfering with the NF-κB pathway along with phosphorylation of AKT and ERK and reduced the expression of calcification markers ALP and Runx2 in osteogenic medium (OM)-induced calcification [63]. These findings suggest that curcumin can be a potential candidate for the prevention of CAVS.

### 4.2. Nobiletin

Nobiletin (5,6,7,8,3′,4′-hexamethoxy flavone) is a dietary polymethoxylated flavonoid found in *citrus* fruits. Nobiletin is reported to have various pharmacological properties, such as cardiovascular protection, anti-inflammatory, antioxidant, anti-cancer, anti osteoclastogenesis, and others [86,87]. Furthermore, nobiletin could reverse the abnormal gene expression profile, delay the process of the aortic valve, and inhibit calcification and phenotypical transformation of hVICs in TNF-α-induced cells by interfering with TNF, PI3K-Akt, mTOR, NF-kappa B, Toll-like receptor pathways [64]. Thus, nobiletin can be a novel target to alleviate VICs calcification.

### 4.3. Caffeic Acid Phenethyl Ester

Caffeic acid phenethyl ester [(phenethyl 3-(3–4 dihydroxyphenyl) acrylate) (CAPE)] is a natural polyphenolic compound found in propolis from honeybee hives and bark of conifer trees [88]. CAPE is known to have potent antioxidant and cytoprotective activities and protective effects against various disease conditions, such as infections, cancer, diabetes, neurodegeneration, and anxiety [89,90,91]. Additionally, CAPE exhibited anti-inflammatory effects by inhibiting the NF-kB pathway [90,91]. Moreover, CAPE reduced the expression of Runx2 and ALP in OM-induced calcification and inhibited NF-kB activation by decreasing phosphorylated IκBα levels in AVICs and interfering with the nuclear translocation of NF-kB p65 [65]. It also inhibited the activation of NLRP3, ASC, P20 and disrupted phosphorylation of AKT and ERK, which are required to promote cell proliferation [65]. All these results indicate that CAPE acts as a negative modulator of hVICs calcification by interfering with the PI3K-AKT, ERK1/2, and NF-κB/ NLRP3 inflammasome pathway.

### 4.4. Celastrol

Celastrol is a triterpene isolated from the *Tripterygium wilfordii* Hook F (TWHF) plant, also known as Lei Gong Teng (Thunder God Vine), which belongs to the family Celastraceae [92,93]. Celastrol is a natural NOX inhibitor that interferes with the interaction between the tandem SH3 domain of p47phox and the proline-rich region of p22phox required for NOX2 activation [57]. Celastrol has been proven to be effective in the treatment of cancer, neurodegeneration [94], obesity, diabetes, inflammations [95], and autoimmune diseases and in preventing atherosclerosis [96]. Celastrol inhibited calcium deposition in VICs, decreased the expression of Runx2 protein and the level of ROS in calcified VICs induced by OM. In addition, it inhibited p-GSK3β and β-catenin expression and also fibronectin and OPN expression levels [57]. Similarly, in an in vivo rabbit model, celastrol reduced NOX2 induction in both calcified AVICs and calcified rabbit valves, preventing the development of aortic valve fibrosis, calcium deposition, and improved cardiac function. Taken together, celastrol alleviated the osteoblast differentiation of VICs in vitro and prevented aortic valve fibrocalcification and stenosis in vivo by inhibiting the NOX2 mediated GSK3β/β-catenin pathway [57]. Celastrol can effectively alleviate VICs differentiation and calcification.

### 4.5. Andrographolide

Andrographolide, a diterpene lactone compound, is the primary component extracted from a Chinese medicinal herb called *Andrographis paniculata* belonging to the family *Acanthaceae*. Andrographolide has been widely used in China and some Asian countries and is commonly called as king of bitters [97]. It has been used extensively to treat various disorders, such as viral infection, diarrhea, dysentery, and fever, for hundreds of years and is also prescribed to treat inflammation-related diseases, such as laryngitis, upper respiratory tract infection, and rheumatoid arthritis, in China [98]. Along with its anti-inflammatory properties, it has a wider range of therapeutic activities, such as anti-tumor, anti-diabetic, and antioxidant [98]. The anti-inflammatory properties of andrographolide inhibit the activation of NF-κB by reducing phosphorylated IKKβ levels [99]. Furthermore, in hVICs, andrographolide reduced osteogenic differentiation of VICs and downregulated the expression of osteogenic markers Runx2 and ALPL or ALP in OM-induced calcification [66]. Moreover, the protein expression of phosphorylated ERK, AKT, IκBα, and NF-κB pathway-related genes was also reduced after andrographolide treatment [66]. These results confirmed the capacity of andrographolide to attenuate calcification by interfering with the AKT, NF-κB, and ERK1/2 signaling pathways, makes it a suitable target for CAVD therapy.

### 4.6. Fucoxanthin

Fucoxanthin is a special xanthophyll found in brown seaweeds, micro and macro algae, which have potent antioxidant activity along with various biological activities, such as anti-inflammatory, antioxidant, anti-tumor, prolonging lifespan, and regulation of lipids metabolism and glycometabolism [100,101]. In addition, fucoxanthin is known to reduce the risk factor of cardiovascular diseases, such as hyperlipidemia, inflammatory markers, hypertension, insulin resistance, and obesity [102]. Furthermore, fucoxanthin inhibited apoptosis by a decreased ROS formation and reduced calcification and extracellular matrix accumulation through phosphorylation regulation of AKT and ERK in H_2_O_2_-induced calcification [67]. Moreover, in an in vivo dog model, fucoxanthin improved compensatory cardiac hypertrophy and valve function [67]. Thus, fucoxanthin can protect human valvular interstitial cells from damage from H_2_O_2_-induced oxidative stress via various mechanisms, including cytoprotective efficacy against H_2_O_2_ and inhibition of Akt/ERK-related signaling pathways.

### 4.7. Cardamonin

Cardamonin (2′,4′-dihydroxy-6′-methoxychalcone) is a naturally occurring chalcone mainly found in different species of the Zingiberaceae family, such as *Ginkgo biloba,* leaves and seeds of *Amomum subulatum,* and others [103,104]. Cardamonin is known to have a potent anti-inflammatory effect that inhibits pro-inflammatory factors, such as nitric oxide, prostaglandin E_2_ [105], and antioxidant activity [106]. Cardamonin is known to prevent calcification of human valvular interstitial cells induced by OM [68]. Cardamonin significantly reduced the expression of various calcification-related protein markers, such as Runx2 and BMP2, along with decreased expression of TNF-α and COL1A2, along with reduced activation of p-AKT, p-ERK1/2, and p-IκBα, and inhibited nuclear transcription of NF-κB p65 [68]. In both in vitro experiments in hVICs and in vivo experiments in ApoE^−/−^ mice, cardamonin exhibited an anti-inflammatory effect and reduced aortic valve calcification by decreasing the activation of NLRP3 inflammasome and reducing IL-1β expression [68]. Thus, cardamonin can be considered as a potential candidate for preventing the progression of CAVS.

### 4.8. Others

In addition to VICs, some studies have investigated the calcification inhibitory effect of antioxidants using vascular smooth muscle cells (VSMCs), a rat model of uremic obesity, and adenine-induced chronic renal failure. The antioxidant compounds used in studies were apocynin [69,70,71], ellagic acid [72], gallic acid [73], puerarin [74,75], silybin [76], quercetin [77], diosgenin [78], vitamin E [79], 10 dehydrogingerdione (10-DHGD) [80], and resveratrol [81].

Apocynin (4-hydroxy-3-methoxyacetophenone) is a plant-derived natural antioxidant found in *Apocynum cannabinum, Picrorhiza kurroa,* and others [107], which is reported to have anti-inflammatory, anti-hypertensive, and vascular injury preventive properties, along with a decrease in cardiac injury and cardiac remodeling [108]. Apocynin reduced the expression of calcification markers, such as BMP2, Runx2, and OPN, along with an increase in contractile marker α-SMA expression in angiotensin II-induced calcification [69]. Additionally, apocynin downregulated the expression of ERK1/2 phosphorylation in VSMCs, thereby inhibiting osteogenic conversion through ERK1/2 pathway regulation [69]. In addition to that, apocynin reduced oxidative stress, differentiation-related markers, and aortic calcification despite high blood glucose levels in the chronic kidney disease rat model [71].

Ellagic acid is a natural polyphenol found in nuts and many fruits, especially red berries, which are a natural antioxidant that scavenges free radicals [72]. Ellagic acid showed antioxidant and lipid peroxidation inhibitory activities. In addition, it has been reported that it improves cardiovascular function and restores endothelial dysfunction in vessels and the heart in animal models and showed anti-proliferative effects in smooth muscle cells, thereby significantly inhibiting arterial calcification in hypertensive rats [72].

Gallic acid (3,4,5-Trihydroxybenzoic acid) is a polyphenolic compound enriched in blackberry and oak barks, and it is known to be effective against melanogenesis inhibition, anti-cancer, anti-ulcerogenic, anti-inflammatory, chronic kidney disease, and high-fat-induced dyslipidemia [73]. Gallic acid has been shown to effectively reduce the mRNA expression of calcification markers, such as Runx2, BMP2, osteocalcin, Msx2, and calcium deposition in VSMCs, along with decreased expression in BMPR1a and BMPR1b mRNA and phosphor- smad1/5/8, confirming the involvement of BMP2 and Smad1/5/8 pathway in calcification-alleviating mechanism of gallic acid in VSMCs [73].

Puerarin (daidzein-8-C glucoside) is a phytoestrogen isolated from *Radix Puerariae* that can be used to treat multiple disorders, such as coronary artery disease, osteoporosis, atherosclerosis, myocardial infarction, and liver fibrosis [75]. Additionally, puerarin inhibited ALP activity, osteocalcin secretion, and Runx2 expression in calcified VSMCs through the ER/PI3K-Akt signaling pathway [75]. In a uremic rat model, puerarin prevented calcium deposition in rat aorta and reduced expression of Runx2 and BMP2. In addition to that, puerarin reduced pro-inflammatory cytokine IL-1β by targeting NLRP3/Caspase1/IL-1β and NF-κB signaling pathways and inhibiting the generation of ROS [74].

Silybin is a natural antioxidant isolated from silymarin and effectively reduced the reactive oxygen species generated in vascular smooth muscle cells calcification [76].

Quercetin (3,3′,4′5,7-pentahydroxyflavone) is a natural flavonoid and a plant secondary metabolite extensively found in fruits and vegetables, such as apples and onions [109]. Quercetin is known to have an inhibitory effect on vascular calcification in adenine-induced chronic renal failure rats through oxidative stress and modulation of the iNOs/p38MAPK pathway [77]. Similarly, quercetin also inhibits oxidized low-density lipoprotein (Ox-LDL)-induced osteogenic differentiation and calcification of VSMCs by the ROS/TLR4 signaling pathway [110].

Diosgenin, a steroidal saponin found in fenugreek and other plants, is known to have anti-inflammatory, anti-proliferative, antioxidant, and lipid peroxidation reducing properties [78]. In chronic renal failure rats, diosgenin reduced calcification in the aorta with a decrease in alkaline phosphatase activity. Additionally, diosgenin decreases the nitric oxide metabolites and also restores enzymatic antioxidant activity [78]. Diosgenin inhibits TNF-α-induced production of intracellular ROS and phosphorylation of p38, ERK, JNK, and AKT and NF-κB activation. These results indicate that diosgenin can reduce calcium deposition and alkaline phosphatase activity and alleviate aortic vascular smooth muscle cells transdifferentiation via the pathways mentioned earlier [78].

Vitamin E, a natural fat soluble antioxidant found in the cell antioxidant defense system, with its most significant form being α-tocopherol, effectively protects cells from oxidation by free radicals [111]. Vitamin E has been reported to relieve various diseases, such as atherosclerosis, oxidative stress, cancer, cataract, Alzheimer’s disease, asthma, allergies, and diabetes [112]. Vitamin E reversed glutathione peroxidase activity, reduced calcium deposition in uremic rats aorta and human vascular smooth muscle cells, and effectively inhibited oxidative stress along with decreased calcium content, bone alkaline phosphatase, and gene expression of core-binding factor-α [79], suggesting vitamin E can alleviate vascular calcification in both uremic rats and human vascular smooth muscle cells.

10-dehydrogingerdione (10-DHGD), found in ginger rhizomes, is proven to inhibit cholesterol ester transfer protein, decreasing LDL-C and increasing HDL-C. Additionally, it has anti-inflammatory and antioxidant activity along with a beneficial effect on coagulation-fibrinolysis homeostasis and nitric oxide release [113,114]. 10-DHGD improved lipid profile in high-cholesterol-fed rabbits, decreased calcium deposition along with reduced expression levels of BMP2 and Wnt3a mRNA [80]. Similarly, it is found to reduce the expression of calcification-related markers, such as OPN, ALP, and osteocalcin. Additionally, 10-DHGD decreased RANK expression with the increase in OPG. It is also found to reduce atherosclerotic lesions in rabbits, implying 10-DHGD can effectively alleviate calcification associated with atherosclerosis [80].

Resveratrol (3,5,4′-trihydroxytrans-stilbene) is a polyphenolic compound commonly found in various types of berries, such as grapes. It is a scavenger of various free radicals and can improve risk factors for diseases such as cancer, diabetes, osteoporosis, and cardiovascular disease [115,116]. Resveratrol can alleviate arterial calcification in ovariectomized rats (preclinical model of post-menopause) mediated by the sirtuin1 (SIRT1) signaling pathway [117]. Resveratrol upregulated the expression of SIRT1, downregulated the expression of calcification markers Runx2, osteocalcin, and ALP, increased OPG along with downregulation of RANKL, and decreased the expression of senescence markers, such as p53, p21, and p16 [117]. Similarly, resveratrol has been reported to effectively inhibit the development of aortic valve calcification and stenosis even in aged mice model (Apoe^−/−^ mice challenged with the Western diet for 16 weeks) [81]. These results indicate resveratrol can be an important candidate for calcification inhibition.

## 5. Conclusions and Future Perspective

CAVS is a progressive disorder with very complex pathophysiology and only treatment option available being the surgical valve replacement or catheter intervention. This treatment option comes with further complications, such as the use of anticoagulants and re-surgery to replace the prosthetic valve. The treatment of CAVS, thus, requires novel potential pharmacotherapies that have not yet been well explored. Many research works are being conducted nowadays to find the potential candidates to reverse or inhibit the progression of CAVS. Since the pathogenesis of CAVS is associated with increased oxidative stress, natural antioxidants can serve as a potential candidate for managing CAVS. However, there are relatively few relevant research articles and few clinical evidence-based studies. With the understanding gained from the current literature, we anticipate that more antioxidants will emerge as potential therapeutic candidates against CAVS in the near future. The clinical efficacy of these natural antioxidants has not yet been determined and should be further explored, along with evaluation of their therapeutic and toxic activity, as well as bioavailability studies to assess the degree of absorption and their kinetics. Thus, further studies should be conducted to determine the clinical efficacy of these natural antioxidants with adequate dose and safety profile for optimal therapies in CAVS.

## Figures and Tables

**Table 1:** The list of antioxidants with their preventive role in CAVS.

Antioxidants	Calcification Inhibition Mechanism	Calcification Model	Reference Number
In vitro experiments conducted in valvular interstitial cells			
Curcumin	Inhibition of NF-κB, AKT, ERK	In vitro; hVIC	[63]
Nobiletin	Inhibition of AKT, NF-κB, TNF-α	In vitro; hVIC	[64]
Caffeic Acid Phenethyl Ester	Inhibition of ERK/AKT/NF-κB/NLRP3 inflammasome pathway	In vitro; hVIC	[65]
Celastrol	Inhibition of NADPH Oxidase 2 and GSK3β/β-catenin pathway	In vitro; porcine AVIC In vivo; rabbit CAVD model	[57]
Andrographolide	Inhibition of NF-κB/Akt/ERK pathway	In vitro; hVIC	[66]
Fucoxanthin	Inhibition of Akt/ERK-related signaling pathway	In vitro; rat heart VIC In vivo; dog model	[67]
Cardamonin	Inhibition of NF-κB/NLRP3 inflammasome pathway	In vitro; hVIC Ex vivo; Human aortic valve leaflet In vivo; ApoE^−/−^ mice model	[68]
In vitro experiments conducted in other vascular cells			
Apocynin	Suppressing extracellular signal-regulated kinase 1/2	In vitro; Vascular smooth muscle cells	[69,70,71]
Ellagic acid	Improving nitric oxide bioavailability and reducing the formation of ROS	In vivo; Rat model	[72]
Gallic acid	Blocking BMP2-SMAD1/5/8 signaling pathway	In vitro; Vascular smooth muscle cell	[73]
Puerarin	NLRP3/CASPASE1/IL-1Β AND NF-ΚB signaling pathways and inhibition of reactive oxygen species ER/PI3K-AKT signal pathway	In vitro; Rat vascular smooth muscle cells, Mice vascular smooth muscle cells In vivo; uremic rats	[74,75]
Silybin	Reducing the formation of ROS	In vitro; Vascular smooth muscle cell	[76]
Quercetin	Oxidative stress and INOS/P38mapk pathway	In vivo; Adenine-induced chronic renal failure rats	[77]
Diosgenin	Reducing the formation of ROS, inhibition of NF-κB/Akt/ERK, p38 pathway	In vivo; Adenine-induced chronic renal failure rats	[78]
Vitamin E	Reducing the formation of ROS	In vivo; Uremic obese rats	[79]
10 dehydrogingerdione (10-DHGD)	HDL-raising effect and attenuation of associated inflammation	In vivo; Rabbit model	[80]
Resveratrol	Mitochondrial ROS inhibition and SIRT1 activation	In vivo; ApoE^−/−^ mice model	[81]
